# Supplementary material for: Association between paracetamol use during pregnancy and perinatal outcomes: Prospective NISAMI cohort
Source: PLoS One. 2022 Apr 18;17(4):e0267270. doi: 10.1371/journal.pone.0267270 (PMC9015137; doi:10.1371/journal.pone.0267270)
Supplement: Attachment — Submitted filename: Reviewer_Responses_Submission.docx [file pone.0267270.s002.docx]

*PLOS ONE*

*March 16, 2022*

**Reviewer responses**

**Comments to the Author**

1. The manuscript is technically sound but there is a great need to review the English language.

**Answer:** As suggested by the reviewer, we revised the manuscript considering the reviewer's comments and we carried out the English proofreading.

2. Limitations have been stated. There is also need to state the strength of the study.

**Answer**: We agree with the reviewer and added the strengths of our study in the paper.

“One of the strengths of this study is the use of a standardized and tested questionnaire, standardization of data collection, and data validation. Another strength is the prospective design of the cohort, in which interviews were conducted during the prenatal period rather than only at delivery.” Page 14, lines 278-281 – section “Strengths and Limitations”.

3. Rationale for sample size has not been stated.

**Answer**: We agree with the reviewer on the need to explain the rationale for our sample size and included this information in the text.

First, we explained the sample size calculation of NISAMI cohort. “The sample size calculation of the NISAMI cohort was previously described [11]. In summary, the sample selection was based on a prevalence of 50%, a precision of 4%; confidence level of 95%, and 10% for possible losses. The minimum sample required to ensure statistical significance and 80% power was 891 pregnant women, yet the total sample included 1,091 women.” Page 4, lines 86-90 – section “Methods”.

Later, we described the total cohort population that originated our study sample. “From June 2012 to February 2014, 1,091 pregnant women met the original study selection criteria. A total of 760 (69.7%) women reported taking medication in the 15 days before the interviews and were included in this study.” Page 7, lines 152-154 – section “Results”.

Finally, we describe the reason why we adopted paracetamol use in the 15 days before the interview as a criterion for inclusion in our analyzes. “Considering that paracetamol can be taken both by prescription and self-medication, consumption could be underestimated due to memory bias and underreporting.” Page 14-15, lines 282-286 – section “Strengths and Limitations”.
